# Anatomic Interactive Atlas of the Loggerhead Sea Turtle (*Caretta caretta*) Coelomic Cavity

**DOI:** 10.3390/ani16050754

**Published:** 2026-02-28

**Authors:** Alberto Arencibia, Aday Melián, Jorge Orós

**Affiliations:** 1 Department of Morphology, Veterinary Faculty, University of Las Palmas de Gran Canaria, Trasmontaña, 35416 Arucas, Las Palmas, Spain; jorge.oros@ulpgc.es; 2 Daydream Software, 35200 Telde, Las Palmas, Spain; adaymc@gmail.com

**Keywords:** interactive atlas, osteology, dissections, computed tomography, magnetic resonance imaging, coelomic cavity, anatomy, loggerhead sea turtle, *Caretta caretta*

## Abstract

The coelomic cavity of sea turtles is affected by congenital, developmental, traumatic, infectious, and organ- or system-specific disorders, making accurate anatomical knowledge essential for veterinary practice. This study presents an open-access, interactive two-dimensional (2D) anatomical atlas of the coelomic cavity of the loggerhead sea turtle (*Caretta caretta*), developed using images obtained from osteology, gross anatomical dissections, computed tomography (CT), and magnetic resonance imaging (MRI). The atlas comprises six osteology images, sixteen anatomical dissection images, eight transverse CT images acquired using bone and soft-tissue windows, six three-dimensional (3D) volume-rendered CT images, and fourteen MRI images (four transverse, five dorsal, and five sagittal), all provided in PNG format. Relevant anatomical structures were segmented and colour-coded for each figure using manual layer-based segmentation software. The Unity 3D platform was employed for image visualisation and assessment, supporting the development of interactive two-dimensional content. This atlas serves as a useful interactive tool for anatomical learning and clinical reference for professionals and students engaged in the conservation of loggerhead sea turtles.

## Simple Summary

Several diseases affect the coelomic cavity of sea turtles, making accurate anatomical knowledge essential. This study presents an open-access, interactive anatomical atlas of the coelomic cavity of the loggerhead sea turtle (*Caretta caretta*). The atlas integrates osteology, anatomical dissections, computed tomography (CT), and magnetic resonance imaging (MRI), with labelled and colour-coded anatomical structures. It includes 50 images and provides an educational resource for veterinarians, biologists, researchers, and students involved in sea turtle conservation.

## 1. Introduction

Investigations into the anatomy, physiology, clinical presentation, and pathology of stranded sea turtles represent a fundamental component of global conservation efforts [1,2,3,4,5,6]. Across the Canary Islands, the loggerhead turtle (*Caretta caretta*) is the sea turtle species most frequently observed. Most individuals originate in the US western Atlantic and arrive via the Gulf Stream, though a notable proportion also derives from Cape Verde, creating a mixed-stock population within the archipelago [7]. At present, the loggerhead turtle is classified as Vulnerable according to the IUCN Red List, with global populations exhibiting a continued decline [8].

Although several extensive reference works on the medicine and surgery of sea turtles and other reptiles have recently become available, providing valuable guidance for veterinarians, veterinary trainees, and technicians involved in sea turtle care [9,10,11,12,13], there remains a clear need for ongoing professional development. Moreover, several authors have advocated for the integration of ‘conservation medicine’ and ‘zoological and wildlife medicine’ into both undergraduate and postgraduate veterinary training [14,15].

A wide range of approaches has been explored to enhance the teaching and learning of veterinary anatomy. The use of live specimens, cadaveric material, traditional dissections, sectional anatomy and plastinated preparations offers valuable support for researchers, students and technical staff engaging with anatomical study [16,17,18,19,20]. Technological progress in computer-assisted learning over the past few years has transformed the way anatomy is taught [21,22].

Image segmentation is an important process in medical imaging, including CT, MRI, and ultrasound, as it divides images into meaningful regions for easier study and visualisation. Automatic segmentation uses computer algorithms to detect patterns in intensity, shape, and position, and can incorporate prior models or artificial intelligence to improve accuracy. Manual layer-based segmentation involves outlining structures or layers by hand and is often used as a reference to validate automatic methods. Both approaches work together to classify and organise tissues according to intensity, morphology, and spatial context, producing hierarchical anatomical representations that enhance structural differentiation and support more accurate clinical analysis [23,24].

Several different applications of segmentation tools have been described as valuable in human medicine using automatic [25,26] and manual [27,28] layer-based segmentation techniques. Although segmentation techniques in veterinary medicine remain limited in development and implementation, certain automatic approaches have demonstrated successful application. Examples include automated segmentations of the ovine, canine, and pig brain [29,30,31]; the canine and equine pelvic limb [32,33]; and the pig skeleton [34]. In addition, manual layer-based segmentation has been employed in veterinary research to delineate anatomical structures with high accuracy, serving as a reference for validating automatic segmentation methods [35,36]. Both approaches enhance anatomical visualisation, improve structural differentiation, and support more precise analyses in veterinary imaging studies. To date, interactive imaging studies in marine turtles have primarily focused on detailed head anatomy using manual layer-based segmentation [37]. However, despite these advances, no studies of the coelomic cavity using manual layer-based segmentation have yet been conducted in this species. The coelomic cavity of sea turtles houses the primary organs of the cardiovascular, respiratory, digestive, hepatobiliary, urogenital, and endocrine systems [16]. The coelomic cavity inherently presents a vulnerability to a wide spectrum of pathological processes, encompassing congenital, developmental, traumatic, infectious, and organ- or system-specific disorders [38,39,40,41,42,43].

A comprehensive understanding of the anatomical features of the coelomic cavity is essential for effective veterinary care and research in sea turtles. In this study, we aimed to develop an interactive anatomical atlas of the coelomic cavity of the loggerhead sea turtle (*Caretta caretta*) using manual layer-based segmentation, integrating data from osteological analysis, gross dissections, CT, and MRI. The resulting atlas offers a high-resolution, reliable reference for anatomical structures, facilitating accurate identification of organs, supporting clinical interventions, and providing a foundation for future studies on functional morphology and conservation biology.

## 2. Materials and Methods

### 2.1. Animals

A total of seven cadavers of loggerhead sea turtles (*Caretta caretta*) (five juvenile females, one subadult male and one young adult female) that had been stranded in the Canary Islands (Spain) and subsequently died during hospitalisation were used for this study. The turtles had been hospitalised at the Tafira Wildlife Rehabilitation Centre (TWRC) (Gran Canaria Island Government) (Las Palmas de Gran Canaria, Spain) due to severe lesions in the head or in the rear and/or front flippers. All cadavers were preserved frozen at −20 °C for one week in the Department of Anatomy at the Veterinary Faculty of Las Palmas de Gran Canaria (Spain). They were subsequently thawed to undergo CT and MRI examinations, perform anatomical dissections, and acquire detailed images of the carapace and plastron bones. Three of the juvenile specimens were used for CT and MRI studies, while the remaining animals were used for anatomical dissections and osteological preparations. The study was approved by the ULPGC’s Animal Experimentation Ethics Committee (CEEA-ULPGC) (File OEBA-ULPGC 02/2016).

### 2.2. CT Technique

Sequential transverse CT scans of the turtles were performed using a 16-slice helical CT scanner (Aquilion, Toshiba Medical Systems, Madrid, Spain). Each specimen was placed symmetrically in ventral recumbency on the examination table, and image acquisition followed a standard clinical protocol (120 kV, 80 mAs; collimation and detector configuration, 16 mm × 0.5 mm; slice thickness, 5 mm; reconstruction interval, 5 mm; acquisition matrix, 512 × 512; helical pitch, 2; and tube rotation time, 0.75 s). This setup enabled the collection of transverse images encompassing the entire coelomic cavity. The raw CT data were archived and analysed using the DICOM workstation OsiriX MD 13.0.2 (Pixmeo, Bernex, Switzerland). To enhance visualisation of coelomic structures, two specific CT window settings were applied: a bone window (window level, 300 HU; window width, 1500 HU) and a soft-tissue window (window level, 60 HU; window width, 400 HU). The DICOM datasets were subsequently post-processed to generate three-dimensional (3D) volume-rendered (VR) reconstructions of the turtles for detailed anatomical assessment.

### 2.3. MRI Technique

MRI examinations were performed on each turtle using a 1.5-T scanner (Signa Excite; General Electric Medical Systems, Madrid, Spain) equipped with an eight-channel human thoracic coil. Throughout the entire MRI procedure, the animals were positioned in ventral recumbency to ensure consistent image orientation. Image acquisition was carried out using a spin-echo pulse sequence. T2-weighted transverse images were obtained with the following parameters: repetition time (TR), 4760 ms; echo time (TE), 107 ms; matrix size, 192 × 192; and slice thickness, 5 mm. Additionally, T2-weighted dorsal sections were acquired under comparable conditions using the following parameters: TR, 4900 ms; TE, 110 ms; matrix size, 192 × 192; and slice thickness, 5 mm. Furthermore, T2-weighted sagittal images were obtained using the following acquisition settings: TR of 4900 ms, TE of 109 ms, a 192 × 192 matrix, and a slice thickness of 10 mm.

### 2.4. Anatomical Dissections and Preparation of Osteological Specimens

Anatomical dissections were performed following previously published necropsy procedures [44], adapted to the anatomical purpose of this study. Internal access to the coelomic cavity was initiated by removing the plastron, which involved sectioning around the margins of the plastral bones and transecting the marginal bridges. Plastron removal also required disarticulating the junctions between both acromial processes and the cranial portion of the plastron. Both front flippers were removed by carefully sectioning the ligamentous attachments between the scapulae and the carapace. The rear flippers and pelvis were left in situ.

After locating the thyroid and thymus, the pericardial sac was opened. The heart was excised by sectioning the proximal portions of the major arteries and veins, along with the gubernaculum cordis connecting the ventricular apex to the caudal region of the pericardial cavity.

The liver, composed of two lobes (the left situated adjacent to the stomach and the larger right housing the gallbladder), was removed by elevating each lobe and transecting its connective attachments. The spleen, an oval-shaped organ situated near the pancreas at the mesenteric border, was excised prior to the removal of the digestive tract. To remove the entire digestive tract, including the pancreas, the ventral aspect of the neck was first exposed. The oesophagus was isolated, ligated cranially with nylon to prevent any backflow of luminal contents, and then carefully freed so that it could be drawn through the space between the two principal bronchi. The stomach was freed by dissecting it away from the left lung. The large intestine was sectioned as far caudally as possible, close to the cloaca, after placing a ligature to prevent the escape of faecal material. At this stage, the urinary bladder, which also opened into the cloaca, became visible.

To dissect both lungs, which are largely attached to the ventral aspect of the carapace, a small incision was made parallel to its longitudinal axis along the lateral margin. The lungs were then carefully freed from their attachments to the ventral carapace using blunt dissection with the fingers. Given that the kidneys are retrocoelomic (located between the coelomic membrane and the carapace), an incision was made in the dorsal coelomic membrane, parallel and lateral to the position of the gonads, after which both kidneys were carefully isolated using blunt dissection with the fingers. Cranio-medially to each kidney, the adrenal glands were identified. The gonads were located cranial to the urinary bladder and caudal to the lungs, exhibiting morphological differences reflective of each specimen’s sexual maturity.

Following anatomical dissections, osteological preparations of the carapace and plastron were produced using standard cleaning protocols designed to preserve the natural morphology and articulation of the bony elements. After the removal of soft tissues, the shell was carefully detached from the underlying viscera and associated musculature. The carapace and plastron were then separated and meticulously cleaned of any residual organic material using fine mechanical instruments under magnification. Once completely free of soft tissue, the skeletal components were thoroughly rinsed with water, degreased, and air-dried under controlled laboratory conditions to prevent deformation and ensure structural integrity.

### 2.5. Anatomic Evaluation

All anatomical dissections, osteological preparations, and corresponding imaging datasets (CT and MRI) were photographed under standardised lighting conditions. The resulting digital images were subsequently processed and refined using image-editing software (Adobe^®^ Photoshop^®^ CS5, Adobe Systems Inc., San Jose, CA, USA) to optimise contrast, sharpness, and overall visual clarity. This integrative methodological approach enabled precise correlation between the transverse and three-dimensional volume-rendered reconstructions obtained from CT and the transverse and dorsal planes derived from MRI, with the corresponding anatomical features documented during dissection and osteological analysis. Anatomical structures of clinical and morphological relevance were identified and labelled in accordance with the internationally recognised *Nomina Anatomica Veterinaria* [16].

### 2.6. Atlas Technical Methods

Building on the use of ITK-SNAP [24] and a previous head atlas of the loggerhead sea turtle (*Caretta caretta*) [37], a unified methodological framework was developed to standardise atlas production. The primary objective was to simplify the atlas creation process while eliminating the need to generate new application builds for each instance. The framework replaced complex segmentation workflows with a structured, layer-based annotation system applied directly on base images.

Manual layer-based segmentation was conducted using software specifically developed for this study by Daydream Software (Telde, Las Palmas, Spain). The methodological approach relied on three key components: a custom layer-creation tool, a strict and reproducible file and folder organisation, and a generic rendering client capable of dynamically loading atlas resources. This configuration ensured reproducibility, facilitated standardised annotation, and reduced the technical overhead associated with atlas generation.

To obtain an accurate and consistent ground truth, a manual, layer-based segmentation protocol with a single annotator was implemented. All manual segmentations were performed by a senior anatomist. The segmentation results were subsequently reviewed by a European Diplomate in Zoological Medicine and Herpetology, as well as by a veterinary radiology specialist from the Veterinary Clinical Hospital of the Faculty of Veterinary Medicine of Las Palmas de Gran Canaria (Spain).

#### 2.6.1. Identification of Common Requirements

An analysis of previously developed atlases was conducted to identify common technical and structural requirements. Based on this analysis, a standardised atlas package specification was defined. Each anatomical structure was stored as an independent PNG image with a fixed resolution of 1024 × 1024 pixels. Images were generated with a transparent background, and the segmented structures were represented using white pixels. A single image was created for each anatomical structure.

A standardised file naming convention was applied (e.g., 01-Structure_Name.png), in which underscores were used instead of spaces and special characters were not permitted. A corresponding JSON file (Labels.txt) was implemented to map internal numeric identifiers to display names, thereby allowing the inclusion of special characters and multilingual text. Each atlas image folder contained several files and parameters that support image visualisation and interaction. A low-resolution preview image (AtlasPreview.png, 256 × 256 pixels) was provided alongside a high-resolution version (FullImage.png, 1024 × 1024 pixels). The file BottomData.txt stores information displayed below the interactive image. Additionally, a zoom parameter was defined as an integer value ranging from 0 to 100, indicating the default zoom level.

The atlas directory was organised according to a strict hierarchical structure to ensure reproducibility and proper referencing. Each atlas type was stored in a separate folder (e.g., 1-Type_Name), and within each type, individual folders were created for each image or subtype. At the root level, a metadata file (InfoAtlas.txt) was included, containing the atlas name, subtitle, left and right credits text, and references to associated media files (ImageLeftCredits.png, ImageRightCredits.png, AtlasCover.png). All folders and files were assigned a unique numeric identifier to enable accurate referencing from JSON data files, and special characters were avoided in folder names to ensure compatibility with server and software environments.

#### 2.6.2. Layer Creation Tool (JavaScript Implementation)

To replace the segmentation workflow previously performed using ITK-SNAP [24], a custom web-based layer creation tool was developed. This tool allowed anatomical structures to be manually segmented on each of the 50 atlas images, without requiring advanced technical skills.

Each atlas package, containing the full-resolution image, the segmentation file, and an accompanying information file, was accessed and verified to ensure that all structure colours were distinct for later identification and that all labels were free of typographical errors. A standardised image resolution was applied (Full HD), and the images were exported in PNG format. All unique structure colours were activated and overlaid with full opacity, producing a Full HD region overlay at the same resolution as the original image.

**Figure 1:**
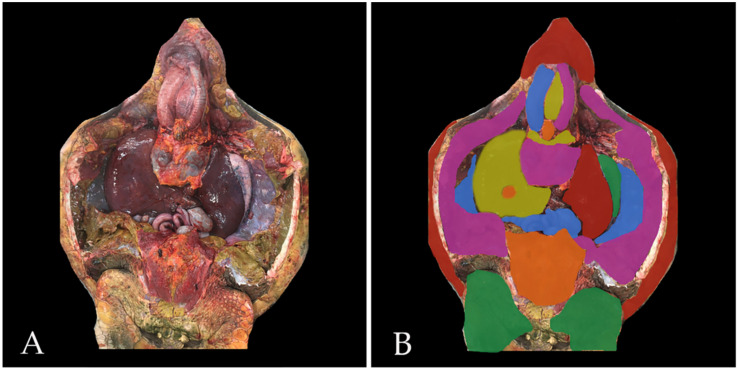
A representative image obtained using manual layer-based segmentation is shown. (**A**): Full-HD-Image.png; (**B**): Full-HD-RegionsOverlay.png.

The information file was exported to support structure identification. All atlas packages were organised within a main folder, subdivided into numbered sub-folders corresponding to each image type (Figure 1), containing the Full HD image, the Full HD regions overlay, and the information file.

##### Web Application Architecture

The tool was implemented in JavaScript using the Konva.js [45] framework. It operates within a standard web browser and was typically executed in a local environment. The application enables full workflow management for atlas editing, including initialisation from any base image, loading and modification of previously exported atlas packages (in .zip format), and progressive editing and refinement of segmentation layers.

##### Layer Creation and Editing

Once a base image or an existing atlas package was loaded, the system allowed the creation of new region layers, the editing of existing layers, and the inclusion of atlas metadata. Anatomical structures could be defined using multiple drawing tools, with polygon-based region painting identified as the most efficient method for rapid segmentation. This approach significantly reduced image preparation time compared to the previous ITK-SNAP workflow.

##### Package Export and Server Upload

After the completion of image annotation and organisation into the required folder structure, the system generated a final atlas package compliant with the standardised format. This package could be uploaded to a designated FTP server configured to serve atlas content through a public API. The API provided structured distribution data, including file paths and download links, enabling dynamic rendering by the atlas client.

#### 2.6.3. Generic Atlas Client

A previously developed Unity WebGL atlas application was adapted into a generic rendering client. The client retrieves atlas data dynamically from a server using a unique atlas identifier. Upon receiving the atlas ID, the client requests a JSON distribution file from the server, parses the folder structure and metadata, and downloads images on demand, caching the retrieved resources locally. Because the application contains no embedded atlas data, it functions as a reusable, content-agnostic rendering engine.

##### Fixed Files Structure Requirements

Strict adherence to the predefined folder and naming conventions was required to ensure compatibility with the rendering client. All folder names and file names must avoid special characters and include numeric prefixes. Root-level metadata files must be present, and consistency between JSON references and file identifiers must be maintained. These constraints ensured reproducibility and prevented server-side incompatibilities.

##### Data Provider: Python API Server

To allow independent management of atlas content, a lightweight server architecture was implemented. The system comprises an FTP server with restricted write permissions and a Python (Version 3.12)-based REST API developed using the Bottle framework.

The API included two main endpoints. The first, the Files Endpoint, received an atlas ID corresponding to a folder name, recursively scanned the directory structure, and returned a JSON file containing file metadata and download links. The second, the Download Endpoint, received a file path parameter and returned the requested file. Cross-Origin Resource Sharing (CORS) policies and SSL certification were configured to ensure secure cross-domain communication.

##### Unity 3D Renderer Adaptation

Unity is a cross-platform engine used for games, simulations, and applications in film, automotive, architecture, and construction [46,47,48]. The Unity WebGL client was modified to remove static references and enable dynamic configuration via URL parameters (e.g., ?atlasId=AtlasName). Upon receiving the atlas ID from the URL, the client requested the distribution JSON from the API, stored the metadata locally, and loaded images on demand during user interaction.

**Figure 2:**
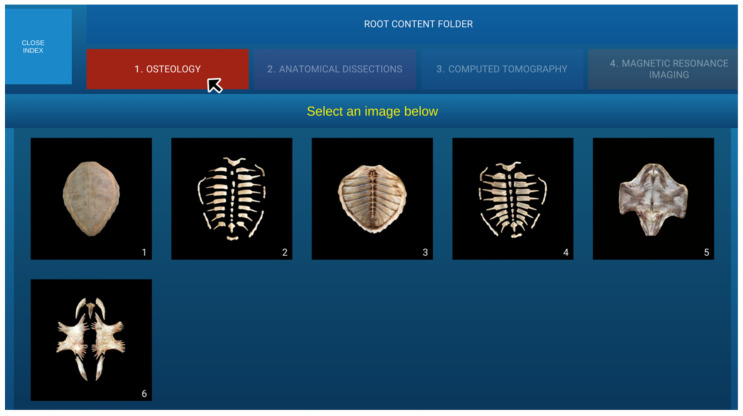
The navigation menu for all atlas sections is shown. When the mouse cursor is placed over a section (Osteology in this case), the section is highlighted in colour, and the corresponding images are displayed.

This architecture allowed a single build to render any compatible atlas without recompilation. A splash screen was implemented with a top menu for selecting different image types, displaying all images from the selected section below (Figure 2).

**Figure 3:**
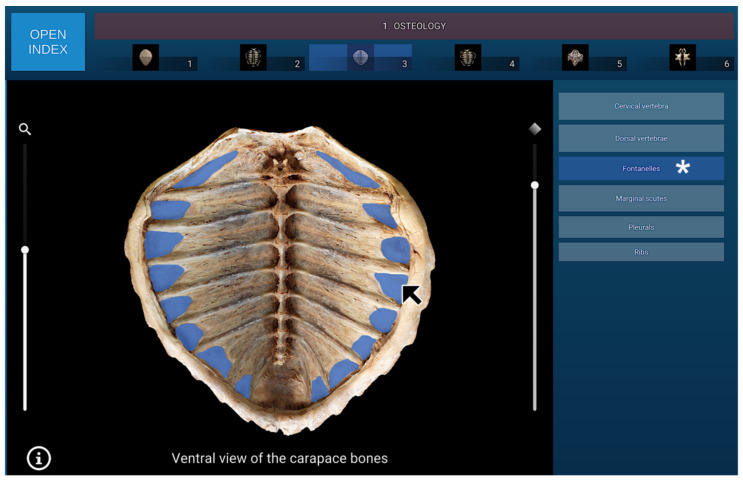
Navigation menu for all labels associated with the osteological image No. 3. When the mouse cursor is placed over an anatomical structure in the selected image, the structure is highlighted in colour, and the corresponding label on the left (Fontanelles in this case) is highlighted simultaneously (white asterisk).

Once an image was selected, the corresponding adapted data were loaded in the Viewer Area, with labels displayed on the right side and the Full HD image on the left (Figure 3).

**Figure 4:**
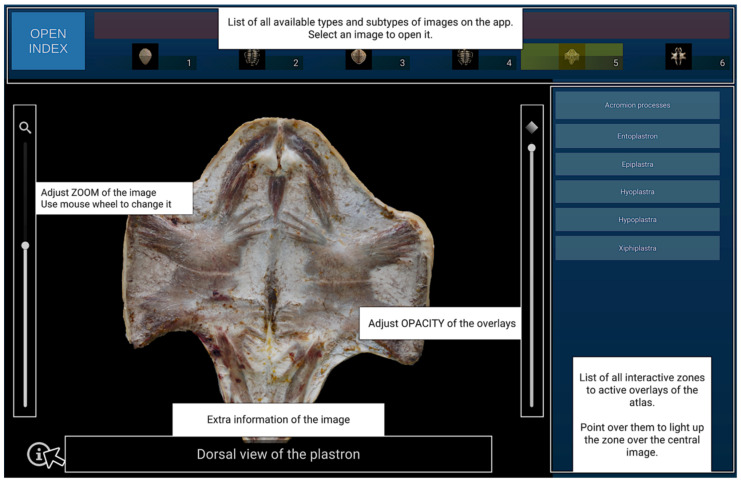
Additional image viewer resources are displayed when the mouse cursor hovers over the information icon.

Additionally, several features were implemented to enhance usability in the image viewer, including zoom, adjustable mask opacity, and an overlay help panel (Figure 4).

##### Local Deployment (Server-Free Configuration)

For the purposes of this study, a server-independent configuration was implemented. In this setup, all resources were stored locally, and a *FilesList.txt* distribution file was embedded within the root folder. A configuration parameter (*isLocal = true*) enabled local file resolution. This adaptation removed external server dependencies while preserving compatibility with the generic rendering system.

The images comprising this atlas are freely accessible at the following website: https://atlascoelomiccavityloggerhead.ulpgc.es/.

## 3. Results

### 3.1. Osteology

The osteology section of the interactive atlas comprises six dynamic visualisations of the turtle’s skeletal system, presented from different anatomical perspectives. Within this section, the bones forming the carapace, including the nuchal, neural, pleural, peripheral, suprapygal, and pygal elements, were identified and labelled. Particular attention was given to the anatomical arrangement of the ribs and dorsal vertebral bodies, as well as to the fontanelles located between the ribs and the carapacial plates.

**Figure 5:**
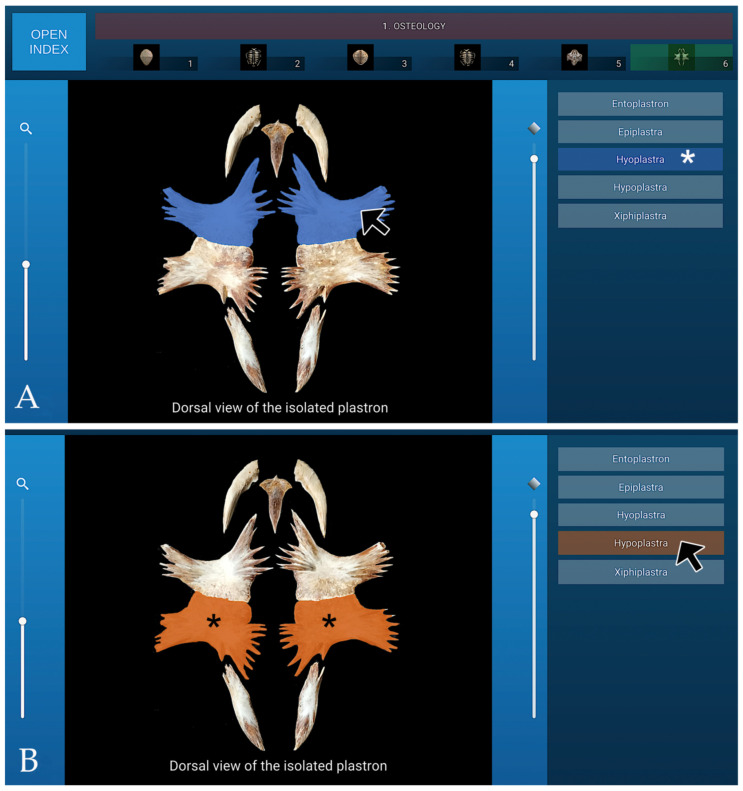
Representative image from the osteology section of the interactive atlas, showing the isolated plastron bones in dorsal view. (**A**): When the cursor is placed over specific bones, they are highlighted in colour, while the corresponding legend identifying the structure (Hyoplastra in this case) is simultaneously highlighted (white asterisk). (**B**): Alternatively, when the cursor is positioned over a specific legend entry (Hypoplastra in this case), the corresponding bones are highlighted in the image (black asterisk).

In addition, the osseous components of the plastron were described in detail, consisting of four paired bones (epiplastra, hyoplastra, hypoplastra, and xiphiplastra) and a single unpaired element (the entoplastron). This section provided a comprehensive visual and structural overview of the morphological integration between the carapace and plastron, serving as a framework for subsequent anatomical and imaging correlations. A representative image from the osteology section of the interactive atlas, illustrating the isolated bones of the turtle’s plastron in dorsal view, is presented in Figure 5.

### 3.2. Anatomical Dissections

The dissection section of the software comprised sixteen high-resolution images that illustrated the principal soft tissues and internal topography of the coelomic cavity. Within these images, the keratinised scutes covering both the carapace and plastron were clearly identified and documented. On the carapace, five distinct types of scutes were observed: the single nuchal scute cranially; five vertebral scutes arranged along the midline; five pairs of costal (pleural) scutes positioned laterally; twelve pairs of marginal scutes bordering the periphery; and a posterior pair of supracaudal scutes overlying the tail base. The plastron displayed the characteristic series of six paired scutes, gular, humeral, pectoral, abdominal, femoral, and anal, arranged symmetrically from cranial to caudal. Together, these features provided a detailed overview of external morphology and served as anatomical landmarks for the identification of underlying skeletal and soft-tissue structures.

During dissections, the walls of the coelomic cavity were clearly identified, together with the thin coelomic membrane lining its internal surface. The dorsal wall was formed by the internal aspect of the carapace and associated connective tissue, while the ventral and lateral walls corresponded to the inner surface of the plastron and adjacent musculature. An abundant amount of adipose tissue was observed throughout the cavity, particularly surrounding the visceral organs and pericardial region. This connective and fatty framework provided a clear anatomical delineation of the coelomic compartment and its spatial relationships with the contained organs.

The axial, pectoral, and pelvic musculature, as well as the muscle groups associated with the cranial and caudal flippers, were generically indicated in the dissection images. These muscular structures were labelled to provide an overall anatomical context; however, no detailed differentiation or individual identification of specific muscles was undertaken, as the primary objective of this section was to illustrate their general distribution and spatial relationships within the coelomic cavity.

The circulatory system was examined with particular attention to the cardiac chambers and the major blood vessels identifiable in the dissection. The heart was clearly distinguishable within the cranial portion of the coelomic cavity and exhibited the three-chambered configuration characteristic of chelonians. The two atria—right and left—and a single ventricle were recognised. From the ventricular region, the principal arterial trunks were observed. The systemic aortae originated dorsally and extended caudally as paired vessels, forming the characteristic systemic arches that eventually merged to create the dorsal aorta. The brachiocephalic trunk was identified as a major branch arising from the arterial outflow tract, giving rise to the subclavian and carotid arteries that supply the forelimbs and cranial regions, respectively. In addition, the pulmonary trunk was clearly visualised, diverging into the paired pulmonary arteries that directed blood flow toward the lungs. Together, these findings allowed a general characterisation of the principal components of cardiovascular anatomy, providing a structural framework for correlating morphological observations with the corresponding tomographic data.

In addition to the main components of the circulatory system, several structures located topographically in close association with the heart were identified. The thyroid gland was observed as a small, bilobed, and highly vascularised organ positioned ventral to the trachea and cranial to the heart, consistent with its typical location in marine turtles. The thymus, although variable in size among individuals, was situated in the cranial region of the coelomic cavity, extending along the ventrolateral aspect of the trachea and great vessels. Both glands were readily distinguishable in the dissection images due to their characteristic coloration and relative position to the cardiac chambers and major arteries. Their identification provided important anatomical context for interpreting the relationships between endocrine and cardiovascular structures within the cranial coelomic compartment, as well as for correlating macroscopic findings with corresponding tomographic features in the imaging datasets. The pericardium and its surrounding connective and adipose tissues were clearly identified in the cranial portion of the coelomic cavity. The heart was enclosed within a well-defined pericardial sac composed of a thin but resistant fibrous membrane that delineated the pericardial cavity and separated the cardiac structures from the adjacent visceral organs. The clear visualisation of these structures in the dissection images provided an accurate anatomical reference for assessing the spatial organisation of the pericardial complex within the cranial coelomic compartment of *Caretta caretta*.

During the dissections, the main components of the digestive system and associated organs were clearly identified within the coelomic cavity. The oesophagus extended caudally from the pharyngeal region, passing along the left side of the trachea before entering the stomach. The stomach was located on the left cranial quadrant of the cavity and exhibited its characteristic J-shaped morphology. Caudally, the pyloric region continued into the small intestine, which was arranged in a series of compact loops occupying the central portion of the coelomic cavity. The large intestine extended caudally toward the cloaca, forming the terminal portion of the digestive tract. The liver was a prominent organ situated in the cranial coelomic region, composed of two lobes—right and left—connected by one or more bands of hepatic parenchyma. The gallbladder was located on the visceral surface of the right hepatic lobe and connected to the duodenum via the bile duct. The pancreas was observed as a flattened, elongated structure closely associated with the duodenal loop, while the spleen appeared as a small, rounded organ attached to the mesenteric border of the intestine. Together, these findings provided a comprehensive topographic overview of the coelomic viscera and their spatial relationships within the cranial and mid-abdominal regions.

The respiratory system was examined to identify its main components, including the trachea, tracheal bifurcation, and primary bronchi. The trachea extended caudally from the glottis and was clearly visible along the ventral aspect of the cervical region, composed of a series of complete cartilaginous rings that maintained a rigid airway. At the level of the thoracic inlet, the trachea was divided into two symmetrical primary bronchi at the tracheal carina. Each bronchus proceeded caudally and dorsally toward its respective lung, penetrating the cranial surface of the paired pulmonary lobes. The lungs were positioned dorsally within the coelomic cavity and attached to the carapace via dense connective tissue septa, consistent with the characteristic structure of chelonians. This organisation of the respiratory tract provided a clear depiction of the airway pathway from the glottis to the lungs, allowing correlation of the macroscopic observations with the corresponding tomographic images for a more comprehensive understanding of the species’ respiratory anatomy.

The main components of the urinary system were identified in the caudal region of the coelomic cavity. The kidneys were paired, elongated, and dorsally positioned organs located within the pelvic region, closely attached to the internal surface of the carapace by dense connective tissue. Each kidney exhibited a dorsoventrally flattened profile and a lobulated surface, features characteristic of marine turtles. The urinary bladder appeared as a thin-walled, sac-like structure situated in the caudoventral portion of the coelomic cavity, ventral to the colon and partially enclosed by peritoneal folds. Its translucent walls and variable degree of distension were evident among the examined specimens.

Both male and female gonads were identified in the caudal portion of the coelomic cavity, located dorsally to the intestines and closely associated with the kidneys. In females, the ovaries appeared as elongated, lobulated structures extending along the dorsolateral aspect of the cavity. The ovarian follicles were clearly visible, varying in size and pigmentation according to the degree of maturity. The oviducts were thin, convoluted tubes that extended cranially from the ovaries, bordered by delicate mesenteric folds. In males, the testes were observed as smooth, elongated organs of uniform colouration, positioned symmetrically on either side of the midline and closely attached to the dorsal body wall.

**Figure 6:**
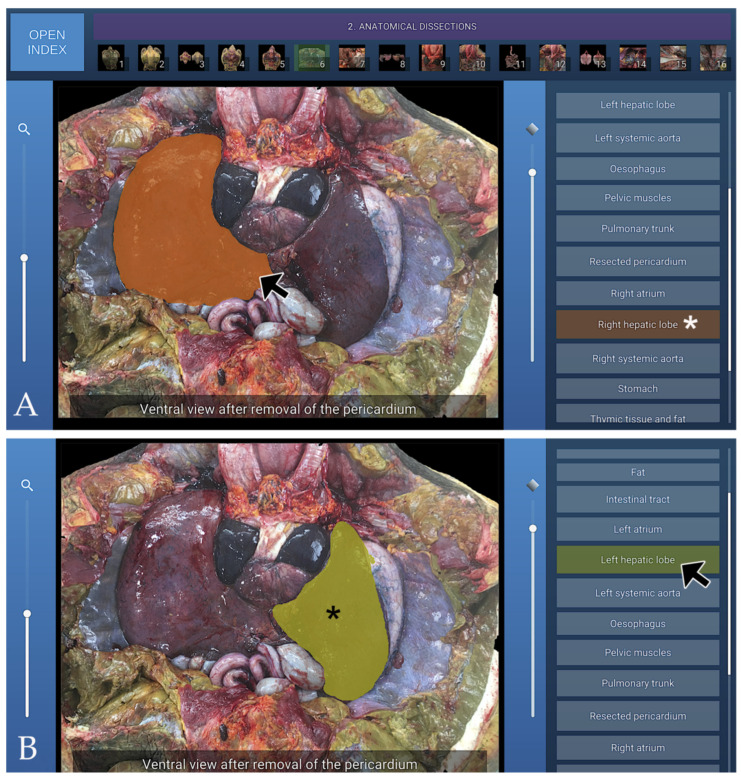
Representative image from the Anatomical Dissections section of the interactive atlas, showing the coelomic cavity in ventral view after removal of the pericardium. (**A**): When the mouse cursor is placed over an anatomical structure, the structure is highlighted in colour, and the corresponding label on the left (Right hepatic lobe in this case) is highlighted simultaneously (white asterisk); (**B**): Alternatively, when the cursor is positioned over a specific legend entry (Left hepatic lobe in this case), the corresponding anatomical structure is highlighted in colour (black asterisk).

A representative image from the anatomical dissections section of the interactive atlas, illustrating the coelomic cavity after removal of the pericardium in ventral view, is shown in Figure 6.

### 3.3. CT Images

The CT section of this atlas comprised three distinct components: transverse images acquired using bone-window (four images) and soft-tissue–window (four images) settings, together with VR reconstructions (six images). The transverse series progressed in a cranial-to-caudal direction, encompassing anatomical regions from the principal bronchi to the kidneys. All images were oriented with the dorsal aspect of the coelomic cavity displayed at the top and the right side shown on the left side of the image.

The 3D VR reconstructions provided complementary three-dimensional perspectives of the skeletal framework and surrounding musculature, particularly within the head, neck, coelomic cavity and flipper regions, serving a skeletal scaffold reference. Two dorsal views were presented, with the right side of the body displayed on the right and the left side on the left. Two ventral views were shown in reverse orientation, with the right side of the body appearing on the left and the left side on the right. Additionally, two left-lateral views were included, with the cranial aspect positioned to the left and the caudal aspect to the right of the image.

Anatomical structures within the coelomic cavity were analysed according to their spatial relationships and attenuation patterns under the respective CT window settings. The evaluation of anatomical structures was conducted differently for the transverse CT images and the VR reconstructions. In the transverse images, structures were analysed according to their spatial relationships and attenuation characteristics in both bone- and soft-tissue–window settings. In contrast, the assessment of the VR reconstructions focused on the three-dimensional morphology, orientation, and topographic relationships of the osseous structures and adjacent musculature, particularly in the cranial, cervical, coelomic cavity, and flipper regions, thereby providing a structural framework for reference, instead of relying on grayscale attenuation values.

#### 3.3.1. CT Bone Window

In cross-sectional imaging of the coelomic cavity of Caretta caretta evaluated using a bone window algorithm, the various anatomical constituents exhibited attenuation patterns that distinctly reflected their structural density and mineral composition. The rigid carapace and plastral elements demonstrated the highest attenuation, appearing as sharply defined, intensely hyperdense cortical margins with a heterogeneous trabecular core typical of chelonian shell architecture. Internal skeletal components, including the fused ribs and pectoral and pelvic girdle elements, similarly displayed marked hyperattenuation, allowing clear delineation from the surrounding soft tissues. Musculature within the coelomic cavity chamber appeared with intermediate attenuation and relatively homogeneous density, while glandular tissues such as the hepatic and exocrine pancreatic regions showed slightly lower, finely heterogeneous attenuation due to their complex lobulated arrangement. Cardiovascular structures, including the ventricle and major arterial trunks, exhibited fluid-equivalent attenuation bordered by thin, mildly hyperdense myocardial walls, whereas the respiratory system, characterised by the presence of air-filled, sac-like lungs, displayed very low attenuation with conspicuous contrast against the surrounding osseous and muscular boundaries.

The urinary system, including the kidneys and urinary bladder, demonstrated mild to intermediate attenuation depending on parenchymal density and luminal fluid content. These gradations in attenuation, enhanced by the high-contrast properties of the bone window, allowed precise visualisation of organ interfaces and structural transitions within the coelomic cavity of *Caretta caretta*, despite the species’ unique fusion of skeletal and shell components.

**Figure 7:**
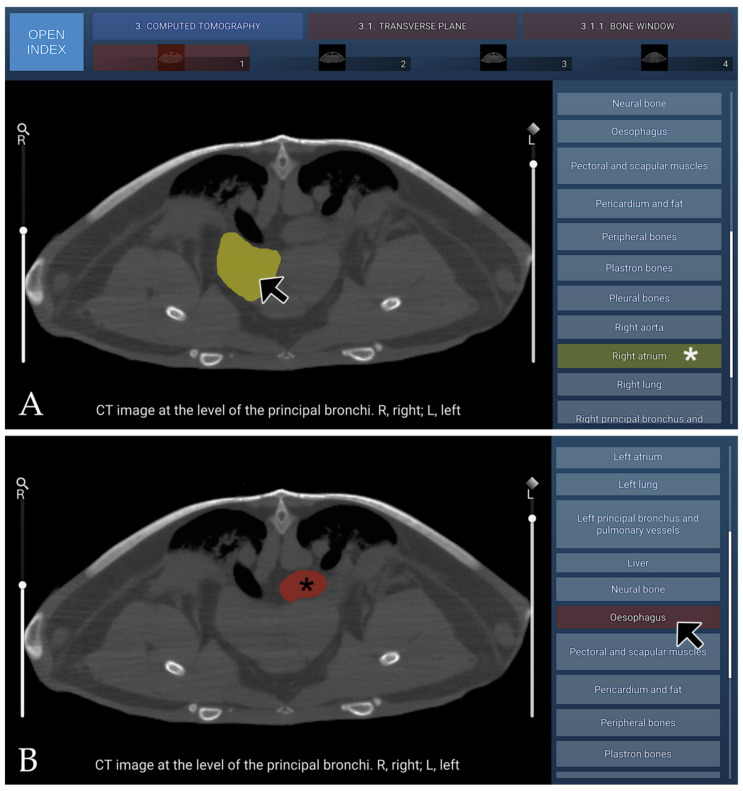
Representative transverse CT image (bone window) at the level of the principal bronchi, shown in cranial view. (**A**) When the mouse cursor is placed over an anatomical structure, the structure is highlighted in colour, and the corresponding label on the left (Right atrium in this case) is highlighted simultaneously (white asterisk). (**B**) Alternatively, when the cursor is positioned over a specific legend entry (Oesophagus in this case), this is highlighted in colour in the image (black asterisk).

A transverse CT image in the bone window at the level of the principal bronchi is presented in Figure 7, as part of the interactive atlas.

#### 3.3.2. CT Soft–Tissue Window

In cross-sectional imaging of the coelomic cavity of *Caretta caretta* assessed with a soft-tissue window algorithm, the attenuation characteristics of internal organs and non-osseous structures were more prominently differentiated, allowing detailed evaluation of their parenchymal composition. Musculature surrounding and traversing the coelomic cavity chamber appeared with intermediate, finely homogeneous attenuation, reflecting the relatively uniform fibre organisation typical of chelonian myology.

Glandular and visceral tissues, including the liver, pancreas, and gastrointestinal components, exhibited lower to intermediate attenuation with subtle internal heterogeneity corresponding to their lobulated and mixed parenchymal architecture.

The cardiovascular system, particularly the ventricle and great vessels, demonstrated fluid-equivalent attenuation within the lumina and slightly higher attenuation in the myocardial walls, which appeared more conspicuous relative to the surrounding soft tissues under these settings.

The respiratory structures, consisting of large, sac-like lungs, showed very low attenuation due to their air content; however, their internal septations, vascular pathways, and membranous boundaries gained definition through enhanced soft-tissue contrast. The renal parenchyma and urinary bladder presented mild to intermediate attenuation depending on internal fluid density and tissue composition, with clearer visualisation of corticomedullary transitions than in bone-optimised windows.

Overall, the soft-tissue window reduced the dominance of the highly attenuating carapace and plastral elements, thereby improving the discernibility of subtle attenuation gradients within the coelomic cavity organs of *Caretta caretta* and enhancing the assessment of their morphology and internal interfaces.

**Figure 8:**
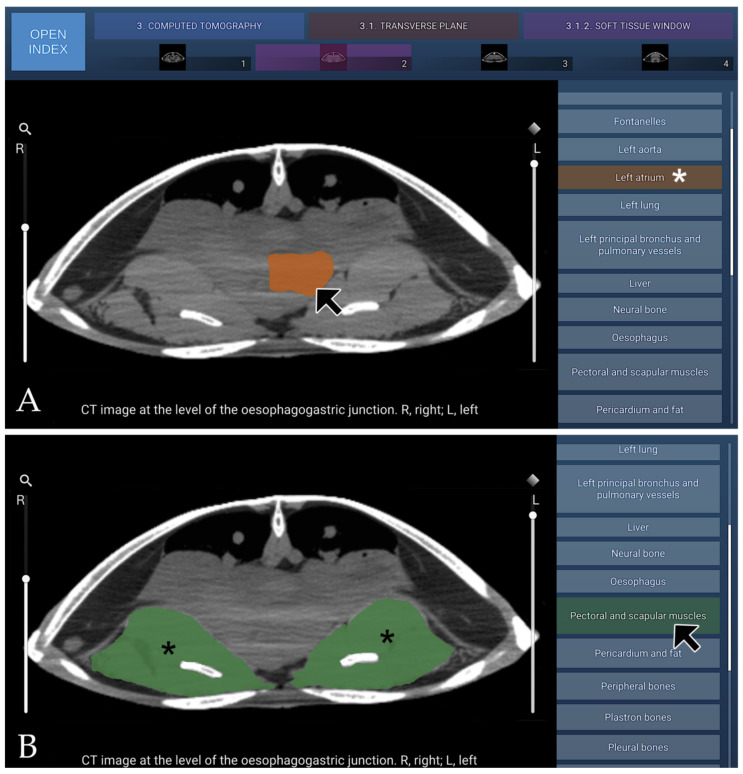
Representative transverse CT image (soft-tissue window) at the level of the oesophagogastric junction, shown in cranial view. (**A**) When the mouse cursor is placed over an anatomical structure, the structure is highlighted in colour, and the corresponding label on the left (Left atrium in this case) is highlighted simultaneously (white asterisk). (**B**) Alternatively, when the cursor is positioned over a specific legend entry (Pectoral and scapular muscles in this case), this is highlighted in colour in the image (black asterisk).

A representative transverse CT image in the soft-tissue window from the atlas, corresponding to the transverse plane at the level of the oesophagogastric junction, is shown in Figure 8.

#### 3.3.3. Three-Dimensional VR Reconstructions

**Figure 9:**
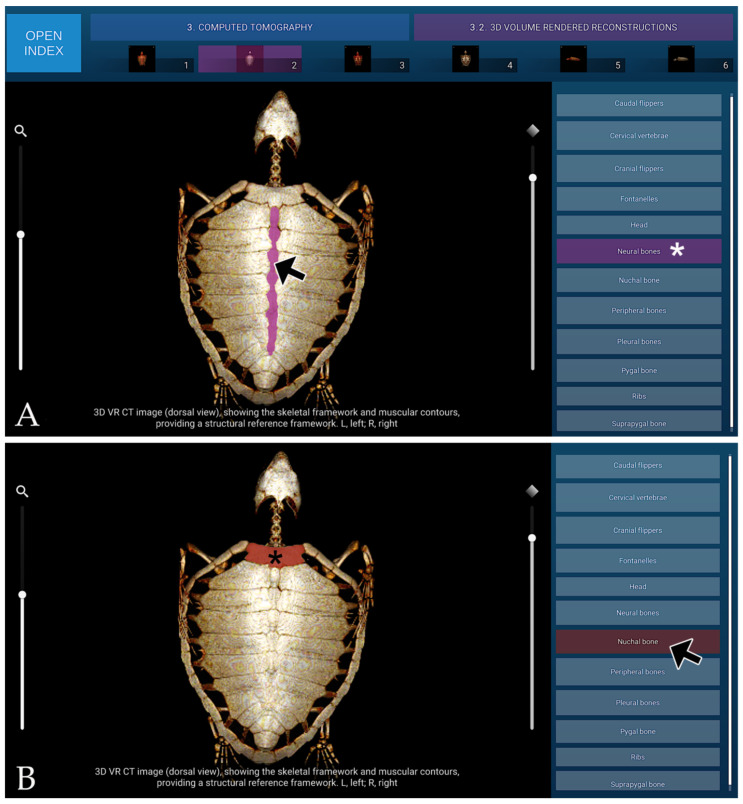
3D VR CT reconstruction (dorsal view), showing skeletal framework and muscular contours, providing a structural reference framework. (**A**) When the mouse cursor is placed over an anatomical structure, the structure is highlighted in colour, and the corresponding label on the left (Neural bones in this case) is highlighted simultaneously (white asterisk). (**B**) Alternatively, when the cursor is positioned over a specific legend entry (Nuchal bone in this case), this is highlighted in colour in the image (black asterisk).

The 3D VR reconstructions enabled clear visualisation of the skeletal framework and surrounding musculature, particularly within the head, neck and flipper regions, providing a structural reference framework. The morphological features and spatial relationships of these structures were well defined. However, internal organs were not visualised due to the absence of contrast enhancement, as no contrast agent had been administered during image acquisition, given the cadaveric nature of the specimens. A representative VR reconstruction CT image from the atlas is shown in Figure 9.

### 3.4. MRI

**Figure 10:**
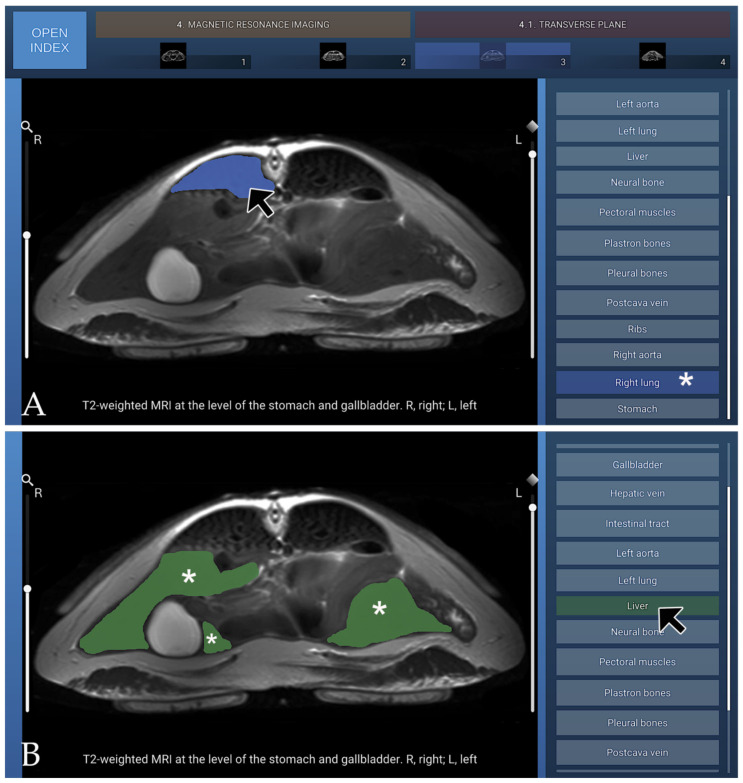
T2-weighted transverse MRI at the level of the stomach and gallbladder, shown in cranial view. (**A**) When the mouse cursor is placed over an anatomical structure, the structure is highlighted in colour, and the corresponding label on the left (Right lung in this case) is highlighted simultaneously (white asterisk). (**B**) Alternatively, when the cursor is positioned over a specific legend entry (Liver in this case), this is highlighted in colour in the image (white asterisk).

**Figure 11:**
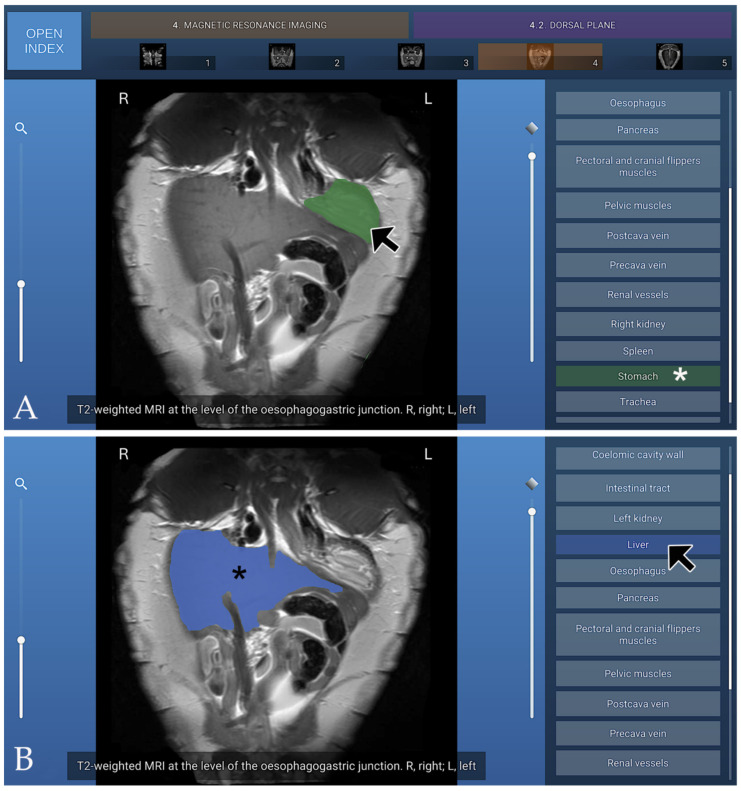
T2-weighted dorsal MRI at the level of the oesophagogastric junction, shown in ventral view. (**A**) When the mouse cursor is placed over an anatomical structure, the structure is highlighted in colour, and the corresponding label on the left (Stomach in this case) is highlighted simultaneously (white asterisk). (**B**) Alternatively, when the cursor is positioned over a specific legend entry (Liver in this case), this is highlighted in colour in the image (black asterisk).

**Figure 12:**
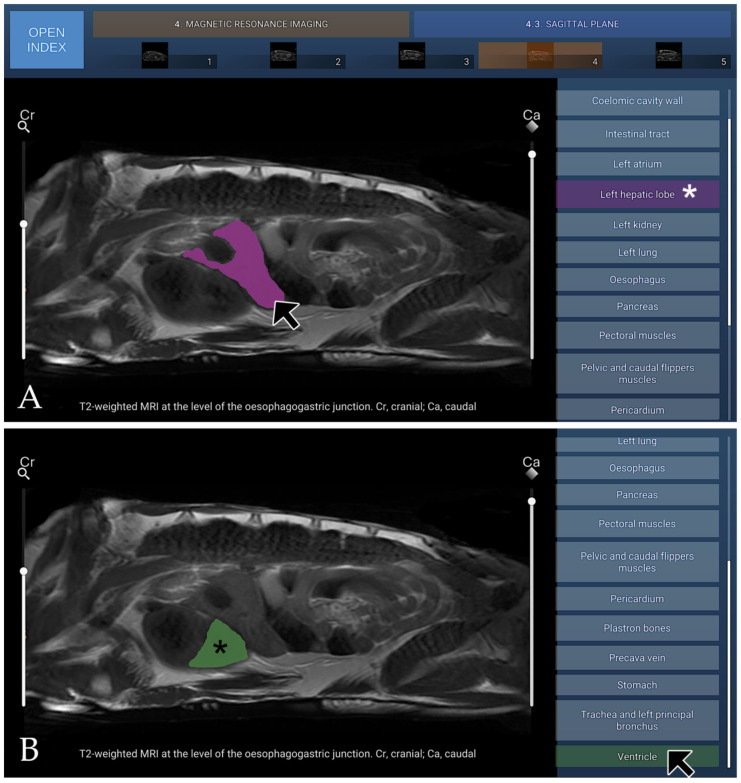
T2-weighted sagittal MRI at the level of the oesophagogastric junction, shown in lateral view. (**A**) When the mouse cursor is placed over an anatomical structure, the structure is highlighted in colour, and the corresponding label on the left (Left hepatic lobe in this case) is highlighted simultaneously (white asterisk). (**B**) Alternatively, when the cursor is positioned over a specific legend entry (Ventricle in this case), this is highlighted in colour in the image (black asterisk).

The MRI section of the atlas includes three anatomical planes—transverse (four images), dorsal (five images), and sagittal (five images)—all acquired using T2-weighted sequences to optimise soft-tissue contrast. Transverse images were arranged in a cranial-to-caudal sequence, extending from the principal bronchi to the kidneys, with the dorsal aspect of the coelomic cavity positioned superiorly and the right side displayed on the left of each image. Dorsal images progressed from ventral to dorsal, beginning at the plastron region and advancing toward the lungs, with the cranial aspect oriented superiorly and the right side appearing on the left. Sagittal images were presented in a lateral-to-medial sequence, extending from the right hepatic lobe and gallbladder to the left hepatic lobe, with the dorsal coelomic cavity surface at the top and the cranial aspect to the left. On T2-weighted MRI, the carapace formed a continuous low-signal band defining the dorsal boundary of the coelomic cavity, bordered internally by a hyperintense layer corresponding to the coelomic membrane and associated fluid. The fontanelles appeared as discrete signal voids consistent with unossified intercostal spaces. In the present cadaveric specimens, the cortical surfaces of the plastral bones exhibited low signal intensity, whereas the medullary regions appeared relatively hyperintense on T2-weighted MRI sequences. The axial, pectoral, pelvic, and cranial and caudal flipper muscle groups demonstrated a uniform intermediate signal intensity, allowing clear differentiation from adjacent soft tissues and facilitating precise anatomical delineation. The trachea was readily identified coursing caudally from the cranial coelom before bifurcating near the cardiac silhouette. Pulmonary parenchyma displayed a heterogeneous but predominantly hyperintense pattern, likely influenced by postmortem and freeze–thaw artefacts affecting air–tissue and vascular interfaces. At the time of imaging, the lungs partially collapsed, which may have contributed to the observed signal characteristics. Bronchial branches appeared as linear hypointense structures within the brighter lung tissue. These features may not fully reflect in vivo pulmonary physiology. The heart was clearly visualised, with both atria and the single ventricle exhibiting well-defined margins and an intermediate soft-tissue signal. Major vessels were easily recognised: the paired systemic aortae arose dorsally from the ventricle, and the brachiocephalic and pulmonary trunks emerged cranially from their respective outflow regions. The subclavian and carotid arteries branched in a typical pattern from the brachiocephalic trunk. The pericardium formed a thin, low-signal envelope that sharply delineated the heart from surrounding coelomic structures. The digestive tract was readily visualised owing to the high signal intensity of luminal fluids on T2-weighted images. The oesophagus extended caudally along the midline, dorsal to the heart, before expanding into a single-chambered stomach located in the left cranial coelom, which appeared as a rounded hyperintense structure surrounded by a thin hypointense muscular wall. The small intestine coursed caudally as a convoluted tubular structure with variable signal intensity, and the large intestine and cloaca occupied the caudal region of the cavity. The liver, one of the largest visceral organs, displayed moderate-to-high signal intensity and a smooth, lobulated contour; the gallbladder appeared as a distinct, spherical hyperintense cystic focus along the right hepatic margin. The pancreas, situated within the duodenal loop, presented an intermediate signal slightly lower than that of the hepatic parenchyma. The overall configuration of the digestive organs reflected the dorsoventral compression imposed by the shell. On T2-weighted MRI, the urinary system was characterised by the high signal intensity of urine within the renal collecting system, ureters, and urinary bladder, consistent with the long T2 relaxation properties of fluid. Renal parenchyma exhibited intermediate signal intensity, with the cortex appearing slightly hyperintense relative to the medulla, while surrounding soft tissues displayed variable intermediate-to-low signal intensities depending on their composition. In both male and female subadult animals, the genital tract was not adequately visualised on T2-weighted sequences. Three T2-weighted MRI figures are presented, corresponding to the transverse plane (Figure 10), the dorsal plane (Figure 11), and the sagittal plane (Figure 12).

A summary of the distinctive features of the anatomical structures present in the loggerhead sea turtle (*Caretta caretta*) in this anatomical, CT, and MRI study is available in the Supplementary Table S1.

## 4. Discussion

The coelomic cavity of sea turtles is particularly interesting because of the location of the principal organs of the cardiovascular, respiratory, digestive, hepatobiliary, urogenital, and endocrine systems [16]. This region constitutes a highly intricate anatomical area, which makes comprehensive physical and clinical evaluation of its structures particularly challenging. Several conditions impacting the coelomic cavity of sea turtles have been identified across studies. Injuries caused by trauma or by the ingestion of foreign bodies may compromise the heart or major blood vessels, resulting in haemopericardium and severe haemorrhage [42]. Severe embolism, with gas bubbles in the cardiac chambers and larger vessels, has been detected in turtles incidentally captured in trawls and gillnets suffering from decompression sickness [42]. In cases of trauma accompanied by intracoelomic haemorrhage, liver injury ought to be a primary concern [38]. In addition to metabolic and nutritional disorders, the liver is susceptible to various microbial and parasitic infections [38,49]. Fibromas in the liver of turtles with fibropapillomatosis have also been reported [50]. Traumatic injuries affecting the digestive system caused by boat strikes are common [40]. Trauma caused by fishhooks and fishing lines is one of the most common causes of stranding in some regions [51]. Ingestion of plastics and other man-made debris can cause obstructions and other complications [52,53]. Recently, intestinal impaction with large amounts of sea urchins has been suggested to be included in the differential diagnosis of gastrointestinal diseases in sea turtles [54]. Pulmonary trauma frequently occurs as a notable complication following boat strikes [39]. Lung infections are frequently observed, and several bacterial and fungal species have been identified in respiratory lesions of sea turtles [49]. Spirorchiid trematodes can parasitise pulmonary blood vessels and embolise ova throughout the lungs [55]. The lungs are a common site for the development of stromal tumours in sea turtles with fibropapillomatosis [56]. The anatomical location of the kidneys results in susceptibility to traumatic injuries caused by boat strikes, explaining the generally poor prognosis for turtles with severe traumatic injuries in the carapace [3]. Gas embolisation within renal vessels has been reported in turtles suffering from decompression sickness [42]. Adrenal glands can be parasitised by spirorchiid trematodes [55]. Embolism of yolk has been reported in sea turtles that died following traumatic events [57].

Advanced imaging modalities such as CT and MRI have become essential tools for the evaluation of the coelomic cavity in sea turtles [58,59]. Due to the rigid bony shell and the compact arrangement of intracoelomic organs, conventional radiography often provides limited diagnostic detail [60]. CT enables high-resolution visualisation of the respiratory system, gastrointestinal tract, hepatic tissue, and reproductive organs, facilitating the detection of coelomic trauma, foreign bodies, pulmonary disorders, and structural abnormalities associated with buoyancy dysfunction [61]. MRI provides superior soft-tissue characterisation [17], particularly for assessing neurological, hepatic, renal, and reproductive tissues. The integration of these non-invasive imaging techniques significantly enhances diagnostic accuracy in the evaluation of coelomic pathology, supports more informed clinical decision-making, and ultimately contributes to improved rehabilitation outcomes in stranded sea turtles [57].

Previous anatomical descriptions of the loggerhead coelomic cavity [60] and lung [5] by CT imaging have provided valuable reference points for the interpretation and characterisation of organ disorders detected on CT. In a study reporting the CT findings in stranded loggerheads, lesions of the skeletal, respiratory, and nervous systems were common, and CT imaging proved valuable for diagnosing trauma in turtles, allowing precise determination of injury extent and facilitating monitoring of the healing process [62]. A combination of neurologic and CT examination has been shown to be beneficial in assessing the clinical significance of carapace deformities and associated neurologic deficits in sea turtles with traumatic injuries [63]. CT imaging also proved useful in evaluating potential spinal cord involvement and guiding decisions regarding reconstructive surgery in stranded turtles presenting with deep, penetrating carapace injuries [64]. Furthermore, CT enabled proactive assessment and facilitated accurate diagnosis of partial intestinal obstruction in a loggerhead undergoing rehabilitation, after conventional radiographs provided inconclusive results [65].

Pulmonary lesions, such as pneumocoelom and granulomatous lesions caused by spirorchiid trematodes infection, have also been diagnosed using CT [6,66,67]. CT has additionally been employed to detect perirenal and cervical gas emboli in loggerheads entrained in hopper dredges [68]. For the early identification of dysbaric osteonecrosis, CT is recommended when conventional radiography fails to demonstrate lesions, particularly in animals with a documented history of decompression sickness and/or joint manifestations [69]. Although comparatively expensive, MRI remains, for some authors, the preferred imaging modality for the assessment of the gastrointestinal tract, as it allows detailed evaluation of soft tissue alterations, particularly those associated with abscess formation and neoplastic processes [70]. MRI has also been used to detect internal tumours in green sea turtles with fibropapillomatosis [71]; in that study, MRI demonstrated more than twice the sensitivity of radiography for the diagnosis of internal fibromas, especially within the lungs. The ability of MRI to identify additional internal visceral tumours varied according to the animal’s overall size, the tumour characteristics, and the contents of the gastrointestinal tract [71].

In the present study, CT provided images of good anatomic quality and good contrast differentiation among tissues in this species. For the preparation of this atlas, a dedicated imaging protocol was applied, employing a wide window setting for bone and a narrow window setting for soft tissues, both of which yielded images of good quality. The bone window settings enabled clear visualisation of the relationship between the cortical and medullary components, whereas the soft-tissue window allowed appropriate differentiation of the major soft-tissue structures. Transverse CT images provided detailed cross-sectional views of the coelomic cavity, enabling precise evaluation of organ morphology and spatial relationships. In addition, VR reconstructions enabled three-dimensional visualisation of the osseous and muscular structures, showing the skeletal framework and muscular contours as a “skeletal scaffold”.

In this study, transverse, sagittal, and dorsal MRI planes were employed to evaluate the coelomic cavity of sea turtles. The transverse plane provided detailed cross-sectional views, allowing accurate assessment of organ morphology and spatial relationships. The sagittal plane offered longitudinal perspectives, enabling visualisation of organ length, overall orientation, and alignment along the cranio-caudal axis. Finally, the dorsal plane facilitated a comprehensive overview of dorsal–ventral structures and interactions among adjacent coelomic cavity organs.

Based on a previous atlas [37], a generic workflow was developed to allow the creation of interactive atlases for animal anatomy using multiple images, adding new depth and meaning through user interaction. The atlas provided interactive, annotated 2D representations of the coelomic cavity, comprising 50 individually segmented images created with manual layer-based segmentation software. This enabled precise identification of anatomical structures, as well as manual tracing and image navigation. Additionally, the resource incorporated colour-coded overlay layers that clearly defined structural boundaries and allowed direct digital labelling of images, eliminating the need for arrows or complex label legends. The atlas is expected to reduce analysis time and increase precision in defining anatomical structures. Manual layer-based segmentation serves as the initial and reliable standard for generating binary masks that could be used in the future to train deep learning models, which is consistent with previous studies in other animal species employing similar layer-based segmentation techniques [35,36]. Manual layer-based segmentation provided correct anatomical delineation but was time-consuming, operator-dependent, and prone to human error, which reduced reproducibility. In comparison, automatic segmentation methods are faster and more consistent, although they may struggle with low-contrast or highly variable structures and often require extensive training data. This approach eliminated the need for ITK-Snap [24] as the image segmentation software, while the layer creation system emulated the process of painting over an image to generate layers, facilitating ease of use.

In this study, visualisation and image analysis were conducted using the Unity 3D real-time development platform [46,47,48], whose various tools enabled the atlas to be built and animated interactively. Unity 3D was selected as the development engine due to its extensive capabilities for creating diverse applications and deploying them across major platforms [46,47,48]. The product was designed to leverage interactive features to explore scientific images, guided by three main principles: easy access from any device, utilisation of authentic scientific data and images, and an intuitive user experience. Subsequent research explored the use of native Javascript and HTML5 solutions as an alternative to Unity 3D, aiming to achieve similar functionality with improved cross-device performance. Integration of the atlas was achieved without the need for third-party servers. The system allowed the atlas to be created and exported to any public web server. An implementation using Javascript and HTML5 was developed while retaining the Unity 3D atlas renderer, which had been employed for several years in veterinary anatomy teaching. Performance remained comparable due to Unity 3D’s excellent WebGL export capabilities.

This study had several limitations that should be considered when interpreting the results. The specimens were juvenile and subadult turtles, representing the age classes typically found in the Canary Islands (Spain), which may have influenced anatomical structures and their appearance on imaging. In subadult males and females, the genital tract was not fully developed and could not be adequately visualised on CT or T2-weighted MRI sequences due to slice thickness, coil limitations, and the absence of a contrast agent. Using adult turtles would likely have improved anatomical identification and image resolution, allowing a more comprehensive depiction of coelomic cavity anatomy. Cadaveric specimens exhibited post-mortem alterations, including ice crystal formation, fluid redistribution, tissue dehydration, post-mortem gas changes, and early decomposition, all of which could influence tissue properties, CT attenuation, MRI signal, and anatomical relationships. Specimens had been frozen at −20 °C prior to imaging, and freeze–thaw processes may have further influenced CT attenuation (e.g., subtle density variations), MRI signals, pulmonary appearance, marrow characteristics, and soft tissue contrast. Pulmonary parenchyma appeared predominantly hyperintense on MRI and showed altered attenuation on CT, likely reflecting partial collapse, fluid accumulation, postmortem redistribution, and freeze–thaw artefacts rather than normal physiology. Bone marrow signal may have reflected postmortem changes and freeze–thaw effects and should not be interpreted as representative of live turtles. On CT, cortical bone appeared hyperdense and medullary regions showed lower density, while on MRI, cortical bone was hypointense and medullary regions relatively hyperintense, reflecting post-mortem changes and sequence parameters.

In vivo, on CT, cortical bone would have been hyperdense and medullary regions would have shown intermediate density, reflecting normal marrow composition with moderate density and homogeneous distribution [60]. On MRI, cortical bone would have been hypointense, and medullary regions would have displayed intermediate signal, with less pronounced hyperintensity and a more homogeneous distribution [17]. These considerations are critical for accurately contextualising the imaging findings when using this atlas as a clinical reference tool.

Technical factors also contributed to limitations. The absence of contrast enhancement restricted vascular differentiation and may have reduced the visibility of subtle soft-tissue interfaces. Image resolution, optimised to balance anatomical detail with web-based performance requirements, may have affected the depiction of very small structures. Segmentation-related factors should also be considered: although manual layer-based segmentation is precise, it is inherently operator-dependent and may be prone to minor interpretation errors, potentially influencing reproducibility and boundary accuracy. Collectively, these limitations, including sample characteristics, cadaveric artefacts, technical constraints, and segmentation factors, may have influenced the visual representation and delineation of certain anatomical structures.

A further limitation of this study was the limited number of individuals involved in validating the interactive atlas. While all validations were performed by highly experienced specialists, the small validator pool may restrict the generalisability of the findings. Nevertheless, they do not compromise the primary objective of providing an accessible and clinically relevant anatomical reference tool. Future studies could incorporate contrast-enhanced imaging, higher-resolution acquisition, and hybrid segmentation approaches to further improve anatomical precision and reproducibility.

However, this interactive atlas provides several advantages over previous studies, which offered early characterisations of the loggerhead coelomic cavity using CT [60] and MRI [17]. The present atlas extends and enhances these findings by combining high-resolution CT and MRI with osteological preparations and anatomical dissections. VR reconstructions allow three-dimensional visualisation of skeletal and muscular structures, providing a more comprehensive depiction of spatial relationships within the coelomic cavity. The interactive format enables dynamic exploration of anatomical structures from multiple planes and angles, offering functionality not available in earlier static publications. Open access ensures broad availability to clinicians, researchers, and educators, supporting enhanced training, clinical decision-making, and comparative studies. Collectively, these features—multimodal integration, interactivity, osteological and dissection-based validation, and broad structural coverage—represent a substantial incremental contribution relative to previous works.

The conservation of sea turtles has become a focus across multiple scientific and academic disciplines, including veterinary medicine [1,2,3,4,5,9,10,11,12,13,14,15,16,17,37,38,39,40,41,42,43,49,50,51,52,53,54,55,56,57,58,59,60,61,62,63,64,65,66,67,68,69,70,71]. The 2D atlas developed in this study provided a novel, accessible, and interactive tool for examining the coelomic cavity of the loggerhead sea turtle. It serves as a practical resource for veterinarians, biologists, researchers, and technicians working in sea turtle conservation at wildlife rehabilitation centres worldwide, supporting the assessment and interpretation of coelomic cavity disorders in this species. Future studies should aim to develop interactive anatomical resources for additional regions of the loggerhead sea turtle, particularly the spinal column and limbs.

## 5. Conclusions

An interactive atlas of the loggerhead sea turtle’s coelomic cavity has been developed by integrating data from osteological analysis, gross dissections, CT, and MRI. This open-access, dynamic resource serves not only as a powerful educational tool for teaching and training in anatomical structures but also provides a valuable reference for clinical assessments, diagnostic imaging interpretation, and research applications. By providing detailed visualisation of coelomic cavity organs and their spatial relationships, the atlas can enhance understanding of both normal anatomy and pathological alterations, supporting improved clinical decision-making, rehabilitation strategies, and scientific investigations in this species.

## Data Availability

The data presented in this study are available at: https://atlascoelomiccavityloggerhead.ulpgc.es/.

## Supplementary Materials

The following supporting information can be downloaded at: https://www.mdpi.com/article/10.3390/ani16050754/s1, Table S1: Distinctive features of the anatomical structures present in the loggerhead sea turtle (*Caretta caretta*) in this anatomical, CT, and MRI study.

## Abbreviations

The following abbreviations are used in this manuscript:

API	Application programming interface
AtlasID	Atlas identifier
CORS	Cross-origin resource sharing
CT	Computed tomography
DICOM	Digital imaging and communications in medicine
FTP	File Transfer Protocol
HTML5	HyperText markup language 5
JSON	JavaScript object notation
MRI	Magnetic resonance imaging
SSL	Secure Sockets Layer
TE	Echo time
TR	Repetition time
URL	Uniform resource locator
VR	Volume rendered
WebGL	Web graphics library
