# Supplementary material for: Anatomic Interactive Atlas of the Loggerhead Sea Turtle (Caretta caretta) Coelomic Cavity
Source: Animals (Basel). 2026 Feb 28;16(5):754. doi: 10.3390/ani16050754 (PMC12985158; doi:10.3390/ani16050754)
Supplement: Supplementary file 1 [file animals-16-00754-s001.zip › Supplementary Table S1.pdf]

Supplementary table

Table S1. Distinctive features of the anatomical structures present in the coelomic cavity of the loggerhead sea turtle (*Caretta caretta*) in this anatomical, computed tomography (CT), and magnetic resonance imaging (MRI) study.

	ANATOMICAL DISECCTIONS	CT BONE-WINDOW	CT SOFT-TISSUE WINDOW	MRI
**Cortical bones (carapace, plastron, ribs)**	Smooth, hard, whitish surface; rigid structures protecting internal organs	Hyperdense, cortical bone very dense, sharp margins	Cortical visible, bone denser than soft tissues	Low signal (hypointense)
**Bone marrow (within long bones and plastron)**	Spongy tissue inside bones	Relatively hypodense compared to cortical bone	Differentiable from cortical bone due to lower density	Relatively hyperintense, possibly influenced by postmortem and freeze–thaw artefacts
**Muscles**	Red-brown, firm, layered around the coelomic cavity	Slightly hyperdense	Soft tissue contrasts visible	Intermediate signal, fibre bundles visible
**Liver**	Right and left lobes, smooth surface, light brown	Marginally hyperdense	Relatively hypodense compared to surrounding parenchyma	Hyperintense, homogeneity variable
**Heart**	Triangular, cranial to liver, ventral to lungs	Hyperdense, defined contours	Slightly hyperdense	Intermediate signal, edges visible
**Stomach**	Muscular sac, left cranial coelomic cavity, reddish	Stomach wall slightly hyperdense	Visible wall	Variable signal

Table S1 (Cont.)

	ANATOMICAL DISECCTIONS	CT BONE-WINDOW	CT SOFT-TISSUE WINDOW	MRI
**Intestines**	Hollow, thin-walled, coiled	Variable hyperdense content	Visible wall	Variable signal
**Pancreas**	Small, elongated, pale tan, along greater curvature of stomach	Slightly hyperdense	Distinguishable as soft tissue structure	Intermediate signal, elongated
**Spleen**	Small, dark brown, medial to stomach	Slightly hyperdense	Hypodense, oval shape	Hyperintense, homogeneous signal
**Lungs**	Spongy tissue, attached to dorsal thoracic wall	Hypodense, alveolar texture hard to differentiate	Hypodense, parenchyma more visible	Hypointense relative to fluid and surrounding fat
**Kidneys**	Paired, elongated, reddish-brown, dorsally retrocoelomic, medial to dorsal muscles, ventral to the vertebrae	Slightly hyperdense, margins visible	Hypodense relative to surrounding tissues, distinguishable from muscles	Intermediate signal, elongated shape visible
**Urinary bladder**	Hollow cavity, thin-walled, oval-shaped, filled with clear fluid	Hypodense	Hypodense, thin wall visible	Hyperintense, fluid content bright
**Ovaries**	Paired, small, oval, pale yellow, located dorsolateral to intestines	Unidentified	Unidentified	Unidentified
**Testicles**	Paired, elongated, pale, medial to kidneys	Unidentified	Unidentified	Unidentified
